# Outcomes of patients with bone metastases from differentiated thyroid cancer

**DOI:** 10.20945/2359-3997000000004

**Published:** 2018-01-01

**Authors:** Inés Califano, Susana Deutsch, Alicia Löwenstein, Carmen Cabezón, Fabián Pitoia

**Affiliations:** 1 Universidad de Buenos Aires Universidad de Buenos Aires Instituto de Oncología A. H. Roffo Service of Endocrinology Ciudad Autónoma de Buenos Aires Argentina Service of Endocrinology, Instituto de Oncología A. H. Roffo, Universidad de Buenos Aires, Ciudad Autónoma de Buenos Aires, Argentina; 2 Hospital General de Agudos J. A. Fernández Endocrine Unit Ciudad Autónoma de Buenos Aires Argentina Endocrine Unit, Hospital General de Agudos J. A. Fernández, Ciudad Autónoma de Buenos Aires, Argentina; 3 Hospital General de Agudos J. M. Ramos Mejía Division of Endocrinology Ciudad Autónoma de Buenos Aires Argentina Division of Endocrinology, Hospital General de Agudos J. M. Ramos Mejía, Ciudad Autónoma de Buenos Aires, Argentina; 4 Hospital Italiano de Buenos Aires Service of Endocrinology and Nuclear Medicine Ciudad Autónoma de Buenos Aires Argentina Service of Endocrinology and Nuclear Medicine, Hospital Italiano de Buenos Aires, Ciudad Autónoma de Buenos Aires, Argentina; 5 Universidad de Buenos Aires Universidad de Buenos Aires Hospital de Clínicas Division of Endocrinology Ciudad Autónoma de Buenos Aires Argentina Division of Endocrinology, Hospital de Clínicas, Universidad de Buenos Aires, Ciudad Autónoma de Buenos Aires, Argentina

**Keywords:** Thyroid cancer, distant metastases, bone metastases

## Abstract

**Objective:**

Bone metastases (BM) from differentiated thyroid cancer (DTC) are associated with poor survival rates. Due to the low frequency of this entity, we performed a multicentric retrospective study that aimed to evaluate the presentation, outcome and causes of death in this population.

**Subjects and methods:**

We reviewed file records from 10 databases. BM were diagnosed by: i) biopsy and/or ii) radioiodine (RAI) bone uptake + elevated thyroglobulin (Tg) levels and/or c) bone uptake of 18-FDG in the PET-CT scan + elevated Tg levels.

**Results:**

Fifty-two patients with DTC were included (44% male, mean age 54 years); 58% had papillary histology. BM were synchronous with DTC diagnosis in 46% of the participating cases. BM were symptomatic in 65% of the cases. Multiple BM were present in 65% of patients, while simultaneous metastatic disease in additional sites was found in 69%. Ninety-eight percent of patients received treatment for the BM, which included RAI therapy in 42 patients; 30 of them received cumulative RAI doses that were larger than 600 mCi ^131^I. The mean follow-up after a BM diagnosis was 34 months. The 2- and 5-year survival rates after diagnosis of the first BM were 64% and 38%, respectively. The status on the last evaluation was DTC-related death in 52% of the patients; 26% of them died from direct complications of BM or their treatments.

**Conclusion:**

BM are usually radioiodine-refractory and are associated with a short overall survival, although most of the patients died of causes not directly related to the BM.

## INTRODUCTION

In most published series, patients with differentiated thyroid cancer (DTC) have a 10-year overall survival rate of 85-93% (1). However, when distant metastases occur, the overall survival may decrease to 50% at 5 years of follow-up (1-4).

Among cases, the most frequent localizations of DTC metastases are the lungs in 50%, bones in 25 to 30% and both sites in 20% (5-8).

The treatment of distant metastases usually involves the use of radioactive iodine (RAI), which may be administered several times during follow-up. Nevertheless, once DTC is no longer amenable to RAI therapy or surgery, the expected survival declines rapidly, and death from thyroid carcinoma within 3 years is common (8,9).

Bone metastases (BM) may severely reduce the quality of life (QoL) in patients with disseminated thyroid cancer, causing pain, fractures and spinal cord compression, among other complications (10). BM is a difficult clinical problem that requires a multidisciplinary approach.

Cancer is generally incurable once it has metastasized to the bone. It has been suggested that bone marrow can serve as reservoir for dormant tumor cells, thereby rendering them resistant to RAI and conventional chemotherapeutic agents (11).

However, the causes of death in patients with DTC and BM have not been widely evaluated, probably due to the low frequency of this entity. Therefore, we decided to perform a retrospective review of patients with DTC and BM that were followed up in 10 referral endocrinology units in Argentina in order to evaluate the epidemiology, forms of presentation, treatments modalities and outcome, including overall survival and causes of death.

## SUBJECTS AND METHODS

We reviewed the file records of patients with DTC whom were treated at 10 hospitals. Since this was a retrospective study, written consent was deemed unnecessary. Confidentiality of patients’ data was maintained in accordance with each institution's standards. We included patients who were treated with total thyroidectomy (either with or without lymph node dissection) and remnant ablation. The patients were classified according to the TNM staging system (AJCC/UICC; 7 ed) (12) and to the risk of recurrence classification from the American Thyroid Association (13). The baseline characteristics of the patients who were included are shown in Table 1.

**Table 1:** The baseline characteristics of 52 patients with bone metastases and differentiated thyroid cancer who were included in the study

Characteristics		Number of patients (%)
Age at diagnosis of thyroid cancer, median (years)		54.5
Range		15-54
> 45 years		42 (80.7)
Male/female		23/29 (44.3/55.7)
**Pathological diagnosis**	
Papillary thyroid cancer		30 (57.6)
	Classic	17
	Follicular variant	9
	Tall cell	3
Oncocytic		1
Follicular thyroid cancer		19 (36.5)
Hürthle cell carcinoma		3 (5.7)
**Stage (AJCC/UICC7ªed)**		
I		4 (8)
II		6 (12.5)
III		7 (15)
IVa		7 (15)
IVb		1 (1.5)
Ivc		23 (48)
**Risk of recurrence (ATA 2009) (n = 47)**	
Intermediate		14 (29.7)
High		33 (70.2)

BM were diagnosed by: i) biopsy and/or ii) after ^131^I bone uptake associated with elevated thyroglobulin (Tg) levels and/or iii) abnormal bone uptake of ^18^fluordeoxyglucose in the positron emission tomography (PET/CT) scan associated with elevated Tg levels.

The following clinical-pathological features of the BM were considered for the analysis: methodology for the diagnosis of the BM, the number and localization of the BM foci, the symptoms generated by the BM, the sites of RAI radioiodine uptake after the administration of the ^131^I dose and the presence of loco-regional disease and/or other non-skeletal metastatic sites.

BM were classified as synchronous if they were detected within the first 6 months after the diagnosis of the DTC and metachronous if they were found later in the follow-up.

### Clinical management during follow-up

After the initial approach, the patients were followed by assessing their clinical status in response to initial therapy by using thyroid hormone withdrawal (THW)-stimulated Tg, along with diagnostic or post-treatment whole body scans (WBS) and neck ultrasonography. All patients underwent morphological imaging, including computed tomography (CT) and/or PET/CT. The response to treatment was defined as follows: i) remission: undetectable stimulated Tg levels with negative post-dose WBS associated to the absence of structural images in CT and or PET/CT); ii) biochemical persistence: detectable Tg levels under thyroid hormone suppressive therapy or after THW, or persistent/increasing levels of TgAb with negative post dose WBS associated to the lack of structural images in CT and/or PET/CT); and iii) incomplete structural response.

Patients with BM received RAI treatments (mean cumulative activity: 391 mCi ^131^I, median 200 mCi, range 100-1500 mCi) until i) adverse events related to RAI appeared or ii) no response to treatment was observed due to progressive disease. Different modalities for the treatment of BM were also evaluated.

We considered the causes of death due to DTC and classified them as secondary to BM when they could be attributed to a direct complication of BM (such as pulmonary thromboembolism caused by immobilization, decubitus ulcer infections in bedridden patients or complications of their treatment, pathological fracture or due to disseminated DTC).

Statistical analyses were performed using SPSS software (version 21: SPSS Inc., Chicago Il). Quantitative variables are expressed as means ± SD; qualitative data are expressed in percentages. Continuous variables were compared using Fisher's exact test and the x^2^ test was used to compare categorical variables. Kaplan-Meier curves are used to present survival times. Values were considered statistically significant at p < 0.05.

## RESULTS

Out of a total of 3810 patients with DTC, 52 (1.3%) were found to have BM and were included in the study (Table 1). BM were diagnosed synchronously (at the moment of the diagnosis of the DTC) in 46.2% of patients (n = 24) and metachronously in 53.8% (n = 28). Metachronous metastases were diagnosed 7-240 months after the initial diagnosis (median: 72 months).

BM were symptomatic in 65.4% of the cases (n = 34). Pain was the most frequent clinical presentation (73.5%, n = 25), while fractures and neurological symptoms were each reported in 8.8% of the cases (n = 3). The characteristics of BM can be observed in Table 2. Either as a presenting symptom or as a later event, spinal cord compression was noted in 6 cases (11,5%).

**Table 2:** Radioiodine uptake, localization and extent of disease in 52 patients with bone metastases from differentiated thyroid cancer

Characteristics		Number of patients (%)
Radioiodine uptake at metastatic site		
	Positive	30 (57.6)
	Negative	22 (42.3)
Number of BM		
	Solitary	18 (34.6)
	Multiple	34 (65.3)
Metastatic site		
	Spine	38 (29.2)
	Pelvis	25 (19.2)
	Ribs	24 (18.4)
	Limbs	19 (14.6)
	Skull	13 (10)
	Clavicle	8 (6.1)
	Sternum	3 (2.3)
Extent of disease at the time of BM diagnosis		
	Isolated BM	12 (23)
	BM+ other metastatic sites	22 (42.3)
	BM+ locoregional disease	4 (7.6)
	BM+ locoregional + other metastases	14 (26.9)

Simultaneous metastatic disease in sites other than bone was found in 69.2% of the patients (n = 36). The lung was the most frequently affected site in 94.4% of the cases (n = 34), followed by the brain, retroperitoneum, adrenal glands and skin (6.6%).

The blood calcium levels were normal in all cases.

Stimulated Tg levels at the time of diagnosis of BM (excluding two patients with positive TgAb) were available in 43 cases. They were < 100 ng/mL in 16% of patients, between 100-1000 ng/mL in 39.5% and > 1000 ng/mL in the remaining 46.5%.

Only one patient was not treated due to advanced disease and poor performance status. The remaining patients received treatment for their BM.

The treatment modalities included RAI therapy in 80.7% (n = 42; 30 of them received cumulative RAI doses larger than 600 mCi ^131^I), intravenous monthly bisphosphonates in 57.6 % (n = 30) and 19 of them received pamidronate, while the remaining 11 were treated with zoledronate, surgery in 50% (n = 26), external beam radiotherapy 50% (n = 26), other therapies in 13.4% (sorafenib n = 4, doxorubicin n = 2, thalidomide and radiofrequency ablation, one patient each).

**Figure 1:**
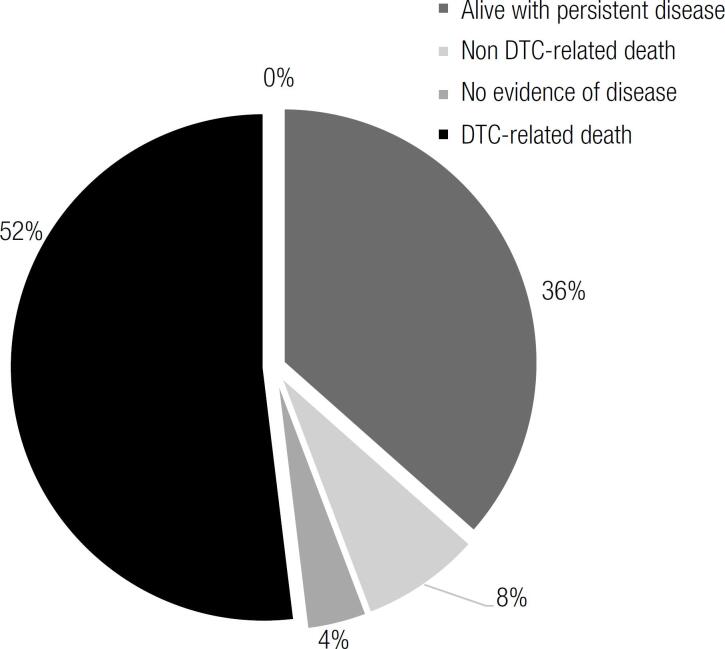
Status of patients with bone metastases from differentiated thyroid cancer at the end of follow-up.

The mean follow-up after diagnosis of first BM was 34.4 ± 33.7 months (median: 24 months, range: 1-67 months). More than 50% of patients died of causes related to the DTC. Hürthle cell carcinoma (30 vs 0%, p 0,005) and fracture as a presenting symptom (20 vs 2.4%, p 0,007) were more frequent in patients who died of DTC-related causes. Radioiodine uptake was found less frequently in patients who died from DTC, although it did not reach statistical significance (40 vs. 61%, p ns). The status on last evaluation can be observed in Figure 1. All of the patients who were alive with persisting disease at the end of the follow-up period had an incomplete structural response.

When DTC-related causes of death (n = 27) were analyzed, 25.9% of the patients (n = 7) died of direct complications of BM or their treatment. The remaining 20 died of other complications related to other localizations of DTC (Table 3).

**Table 3:** Causes of death in 27 patients with bone metastases from differentiated thyroid cancer

Cause of death		Number of patients (%)
Non-BM related		20 (74.1)
	Respiratory insufficiency	11
	Airway obstruction	2
	Central nervous system progression	2
	Sepsis	2
	Treatment complications	1
	Other	4
Secondary to complications of BM		7 (25.9)
	Pulmonary thromboembolism	3
	Sepsis	1
	Treatment complications	1
	Other	2

**Figure 2:**
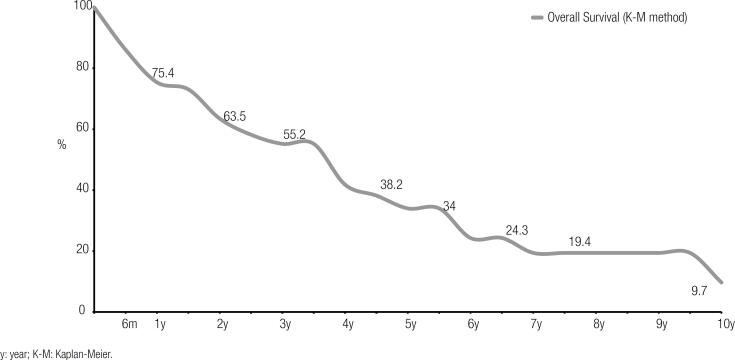
Overall survival after the development of the first bone metastasis in patients with differentiated thyroid cancer (n = 52).

The 2- and 5-year survival rates after the diagnosis of the first BM were 63.5% and 38%, respectively; the overall survival is depicted by the Kaplan-Meier curve in Figure 2.

The median overall survival was 24 months (range 1-120 months).

When we analyzed mortality related to BM (Table 4), the presence of papillary thyroid cancer and painful BM were more frequent in patients who died from causes not related to the BM.

**Table 4:** Analysis of variables related to mortality in patients who died from bone metastasis compared to those who died from other causes

		Death related to BM (n = 10)	Death related to other causes (n = 19)	P
< 45 years old		2 (20%)	4 (21.1%)	NS
≥ 45 years old		8 (80%)	15 (78.9%)	
Female		4 (40%)	9 (47.4%)	NS
Male		6 (60%)	10 (52.6%)	
Histology				
	Papillary	3 (30%)	15 (78.9%)	0.02
	Follicular	4 (40%)	4 (21.1%)	NS
	Hürthle	3 (30%)	-	0.003
Synchronous		5 (50%)	6 (31.6%)	NS
Metachronous		4 (40%)	13 (68.4%)	
Asymptomatic		4 (40%)	7 (36.8%)	NS
Pain		1 (10%)	10 (52.6%)	0.03
Fracture		2 (20%)	1 (5.3%)	NS
Tumor		2 (20%)	-	
Other		1 (10%)	1 (5.3%)	NS
Positive RAI uptake		4 (40%)	14 (73.7%)	0.08
Solitary		4 (40%)	4 (21.1%)	NS
Multiple		6 (60%)	15 (78.9%)	

RAI: radioiodine; NS: non-significant.

## DISCUSSION

Local or distant metastases occur in nearly 10% of patients with DTC (14). In this setting, therapeutic options may include the use of RAI, surgery, and/or the use of external beam radiotherapy (EBRT), among others (15). Nevertheless, between one-third to twothirds of patients with metastatic DTC will become RAI refractory, and this situation is often seen in patients with BM (8). As we observed in our study, most patients fulfill the current criteria for RAI refractoriness (16).

This subgroup of patients generally has a poor overall prognosis, with 10-year survival rates of only 10% and median survival from the discovery of metastases of only 3 to 5 years (8).

DTC has a high tendency to metastasize to bone compared to other tumors; it was reported to be the third most frequent solid tumor after breast and prostate cancer (17), although the physiopathology of BM from DTC is largely uncharacterized.

Follicular thyroid cancer and papillary thyroid cancer show different patterns of spread. The former has a higher propensity to disseminate to bones (7-28% versus 1.4-7% for with papillary thyroid cancer), probably due to a higher frequency of haematogenous dissemination (10,18). However, in absolute terms, in our study we found a greater number of patients with papillary carcinoma and BM, similar to that reported in other series (19). This finding is probably related to the overall higher incidence of papillary thyroid cancer compared to follicular thyroid cancer that, in most current series, comprises only 4 to 7% of the cases.

Skeletal related events (SRE), such as pathological fractures, spinal cord compression, pain and hypercalcemia, are frequent events that adversely affect the QoL of DTC patients with BM (20). As expected, in the present series, 65% of the patients presented symptoms related to BM.

Most of the BM are located in the spine (21) and, as a consequence, spinal cord compression may occur in 14 to 50% of the cases (22,23). In the present series, it was found in 11% of the cases.

Nearly half of the patients in our study presented with pain as the initial symptom. Osseous pain was more frequent in those patients who died due to causes not related to BM (52.6% vs. 10%). It is possible that pain may have led to an earlier BM diagnosis before severe complications occur.

The incidence of hypercalcemia in BM from DTC is variable and has been reported to be as low as 0-4% (19,20,23) to 25-37% (24). We found no cases of hypercalcemia in the present series. Tumoral hypercalcemia is mainly caused by parathyroid-hormone-related peptide secretion (25). Nevertheless, the physiopathology of hypercalcemia occurring in patients with DTC remains to be elucidated.

Considering the treatment modalities, a combination of multiple strategies is generally recommended for patients with BM from DTC (19). Nearly 90% of our patients received more than one option of treatment. In addition to ^131^I therapy (81%), surgery (50%), EBRT (50%) and bisphophonates (60%) were frequently used. In selected cases, chemotherapy, sorafenib and radiofrequency ablation were also indicated.

RAI was used in 81% of patients in our series; 60% of them showed uptake of ^131^I in BM. However, only one patient achieved remission after RAI as the only modality of treatment (cumulative activity of 400 mCi ^131^I). Sabra and cols. demonstrated the lack of oncological benefit in the majority of patients treated with I-131 for distant metastases (9), highlighting that RAI therapy is rarely curative in these patients when used as a solitary therapy. However, an impact on the survival of patients treated with RAI was shown by some authors (19,22,26). Moreover, the complete surgical resection approach followed by RAI therapy has been related to better overall survival (19,27-29).

The complete resection of BM can rarely be achieved when bone disease comprises multiple sites (as was the case in 63% of our series) and the palliative resection of BM has less of an impact on survival (84.4% vs. 55.3%) (29). It should only be considered to obtain a better QoL (30). The mean 5-year survival in DTC patients with BM ranges from 41% to 87% (20,26,28,31). Accordingly, we found that 38% of patients in our series were alive 5 years after the diagnosis of the first BM.

Prognostic factors for cancer-specific mortality in patients with distant metastases from DTC have been widely studied (8,32-34). Most series consistently report that follicular carcinoma, poorly differentiated histology, a lack of radioiodine uptake, advanced age, widespread disease and BM *per se* are predictors of a worse prognosis and shorter overall survival. Poorly differentiated thyroid cancer patients were excluded from the present study; however, all of the remaining risk factors were found in the majority of our population. In this setting, other metastatic sites (such as the lung) or local recurrences are often the immediate causes of death. In the study by Kitamura and cols. (32), the causes of death were respiratory insufficiency (43%), circulatory failure (15%), hemorrhage (15%) and airway obstruction (13%). Nevertheless, in some cases it was not possible to identify the specific cause, due to the simultaneous compromise of several organs.

In the existing literature, the rates of mortality due exclusively to BM are variable and they are usually low (0 to 24%) (6,35,36). In our study, less than one-third of the deaths were directly related to BM. In the remaining cases, death was caused by other complications of advanced DTC, mainly related to respiratory events.

In conclusion, to our knowledge, this is the first multicentric study performed in Latin America on this topic. BM cause significant morbidity and should be managed with a multidisciplinary approach that aims to improve the QoL. Patient selection is important to tailor therapy to individual needs. Further studies with bone targeted agents are needed to assess the utility of these therapeutic modalities in DTC patients. BM are usually RAI refractory and are associated with a decrease in overall survival, although the causes of death are mainly related to complications of non-BM localizations of DTC.

## References

[1] 1. Hundahl SA, Fleming ID, Fremgen AM, Menck HR. A National Cancer Data Base report on 53,856 cases of thyroid carcinoma treated in the U.S., 1985-1995. Cancer. 1998;83(12):2638-48. 10.1002/(sici)1097-0142(19981215)83:12<2638::aid-cncr31>3.0.co;2-1 9874472

[2] 2. Ruegemer JJ, Hay ID, Bergstralh EJ, Ryan JJ, Offord KP, Gorman CA. Distant metastases in differentiated thyroid carcinoma: a multivariate analysis of prognostic variables. J Clin Endocrinol Metab. 1988;67(3):501-8. 10.1210/jcem-67-3-501 3410936

[3] 3. Hoie J, Stenwig AE, Kullmann G, Lindegaard M. Distant metastases in papillary thyroid cancer. A review of 91 patients. Cancer. 1988;61:1-6. 10.1002/1097-0142(19880101)61:1<1::aid-cncr2820610102>3.0.co;2-r 3334935

[4] 4. Tickoo SK, Pittas AG, Adler M, Fazzari M, Larson SM, Robbins RJ, et al. Bone metastases from thyroid carcinoma: a histopathologic study with clinical correlates. Arch Pathol Lab Med. 2000;124(10):1440-7. 10.5858/2000-124-1440-BMFTC 11035572

[5] 5. Schlumberger M, Tubiana M, De Vathaire F, Hill C, Gardet P, Travagli JP, et al. Long-term results of treatment of 238 patients with lung and bone metastases from differentiated thyroid carcinoma. J Clin Endocrinol Metab. 1986;63(4):960-67. 10.1210/jcem-63-4-960 3745409

[6] 6. Samaan NA, Schultz PN, Hickey RC, Goepfert H, Haynie TP, Johnston DA, et al. The results of various modalities of treatment of well differentiated thyroid carcinomas: a retrospective review of 1599 patients. J Clin Endocrinol Metab. 1992;75(3):714-20. 10.1210/jcem.75.3.1517360 1517360

[7] 7. Mazzaferri E. Thyroid carcinoma; papillary and follicular. In: Mazzaferri E, Saaman NA. Endocrine Tumors. Cambridge, MA: Blackwell Scientific; 1993. p. 278-333.

[8] 8. Durante C, Haddy N, Baudin E, Leboulleux S, Hartl D, Travagli JP, et al. Long term outcome of 444 patients with distant metastases from papillary and follicular thyroid carcinoma: benefits and limits of radioiodine therapy. J Clin Endocrinol Metab. 2006;91(8): 2892-9. 10.1210/jc.2005-2838 16684830

[9] 9. Sabra MM, Dominguez JM, Grewal RK, Larson SM, Ghossein RA, Tuttle RM, et al. Clinical outcomes and molecular profile of differentiated thyroid cancers with radioiodine-avid distant metastases. J Clin Endocrinol Metab. 2013;98(5):E829-36. 10.1210/jc.2012-3933 PMC3644606 23533233

[10] 10. Wexler JA. Approach to the thyroid cancer patient with bone metastases. J Clin Endocrinol Metab. 2011;96(8):2296-307. 10.1210/jc.2010-1996 21816796

[11] 11. Weilbaecher KN, Guise TA, McCauley LK. Cancer to bone: a fatal attraction. Nat Reviews Cancer. 2011;11:411-25. 10.1038/nrc3055 PMC3666847 21593787

[12] 12. Edge S, Byrd D, Compton C, Fritz AG, Greene FL, Trotti A. Thyroid. In: American Joint Committee Cancer Staging Manual TNM Classsification 7Th Ed. New York, NY: Springer; 2010. p. 87-96.

[13] 13. Cooper DS, Doherty GM, Haugen BR, Kloss RT, Lee SL, Mandel SJ, et al. Revised American Thyroid Association management guidelines for patients with thyroid nodules and differentiated thyroid cancer. Thyroid. 2009;19:1167-214. 10.1089/thy.2009.0110 19860577

[14] 14. Muresan MM, Olivier P, Leclere J, Sirveaux F, Brunaud L, Klein M, et al. Bone metastases from differentiated thyroid carcinoma. Endocr Relat Cancer. 2008;15:37-49. 10.1677/ERC-07-0229 18310274

[15] 15. Haugen BR, Alexander EK, Bible KC, Doherty GM, Mandel SJ, Nikiforov YE, et al. 2015 American Thyroid Association management Guidelines for adult patients with thyroid nodules and differentiated thyroid cancer. Thyroid. 2016;26(1):1-133. 10.1089/thy.2015.0020 PMC4739132 26462967

[16] 16. Schlumberger M, Brose M, Elisei R, Leboulleux S, Luster M, Pitoia F, et al. Definition and management of radioactive iodine-refractory differentiated thyroid cancer. Lancet Diabetes Endocrinol. 2014;2(5):356-8. 10.1016/S2213-8587(13)70215-8 24795243

[17] 17. Coleman R. Clinical features of metastatic bone disease and risk of skeletal morbidity. Clin Cancer Res. 2006;12 (20 suppl):6243-9. 10.1158/1078-0432.CCR-06-0931 17062708

[18] 18. Kuschayeva YS, Kuschayev SV, Carroll NM, Felger EA, Links TP, Teytelboym OM, et al. Spinal metastases due to thyroid carcinoma: an analysis of 202 patients. Thyroid. 2014;24(10):1488-500. 10.1089/thy.2013.0633 24921429

[19] 19. Orita Y, Sugitani I, Matsuura M, Ushijima M, Tsukahara K, Fujimoto Y, et al. Prognostic factors and the therapeutic strategy for patients with bone metastasis from differentiated thyroid carcinoma. Surgery. 2010;147:424-31. 10.1016/j.surg.2009.10.009 20176243

[20] 20. Farooki A, Leung V, Tala H, Tuttle RM. Skeletal-related events due to bone metastases from differentiated thyroid cancer. J Clin Endocrinol Metab. 2012;97(7):2433-9. 10.1210/jc.2012-1169 22564664

[21] 21. Zhang W, Liu D, Feng C, Zhou C, Zhan C, Que R, et al. Management of differentiated thyroid carcinoma with bone metastasis: a case report and review of the Chinese literature. J Zhejiang Univ Sci B. 2014;15(12):1081-7. 10.1631/jzus.B1400124 PMC4265563 25471838

[22] 22. Pittas AG, Adler M, Fazzari M, Tickoo S, Rosai J, Larson SM, et al. Bone metastases from thyroid carcinoma: clinical characteristics and prognostic variables in one hundred forty-six patients. Thyroid. 2000;10(3):261-8. 10.1089/thy.2000.10.261 10779141

[23] 23. Orita Y, Sugitani I, Takao S, Toda K, Manabe J, Miyata S. Prospective evaluation of zoledronic acid in the treatment of bone metastases from differentiated thyroid carcinoma. Ann Surg Oncol. 2015;22(12):4008-13. 10.1245/s10434-015-4497-0 25762482

[24] 24. Wu K, Hou S, Huang T, Yang R. Thyroid carcinoma with bone metastases: a prognostic factor study. Clin Med Oncol. 2008;2:129-34. 10.4137/cmo.s333 PMC3161655 21892275

[25] 25. Mirrakhimov A. Hypercalcemia of malignancy: an update on pathogenesis and management. N Am J Med Sci. 2015;7:483-93. 10.4103/1947-2714.170600 PMC4683803 26713296

[26] 26. Bernier MO, Leenhardt L, Hoang K, Aurengo A, Mary JY, Menegaux F, et al. Survival and therapeutic modalities in patients with bone metastases of differentiated thyroid carcinoma. J Clin Endocrinol Metab. 2001;86(4):1568-73. 10.1210/jcem.86.4.7390 11297585

[27] 27. Zettinig G, Fueger BJ, Passler C, Kaserer K, Pirich C, Dudczak R, et al. Long-term follow-up of patients with bone metastases from differentiated thyroid carcinoma – surgery or conventional therapy? Clin Endocrinol (Oxf). 2002;56(3):377-82. 10.1046/j.1365-2265.2002.01482.x 11940050

[28] 28. Qiu Z-L, Song H-j, Xu Y-Hong, Luo Q-Y. Efficacy and survival analysis of I131 therapy for bone metastases from differentiated thyroid cancer. J Clin Endocrinol Metab. 2011;96(10):3078-86. 10.1210/jc.2011-0093 21795449

[29] 29. Nakayama R, Horiuchi K, Susa M, Watanabe I, Watanabe K, Tsuji T, et al. Clinical outcome after bone metastasis surgery in patients with differentiated thyroid carcinoma: a retrospective study of 40 cases. Jpn J Clin Oncol. 2014;44(10):918-25. 10.1093/jjco/hyu099 25104791

[30] 30. George R, Jeba J, Ramkumar G, Chacko AG, Tharyan P. Interventions for the treatment of metastatic extradural spinal cord compression in adults. Cochrane Database Syst Rev. 2015;(9):CD006716. 10.1002/14651858.CD006716.pub3 PMC6513178 26337716

[31] 31. Do M, Rhee Y, Kim D, Kim C, Nam K, Ahn C, et al. Clinical features of bone metastasis resulting from thyroid cancer: a review of 28 patients over a 20-year period. Endocr J. 2005;52(6):701-7. 10.1507/endocrj.52.701 16410661

[32] 32. Kitamura Y, Shimizu K, Nagahama M, Sugino K, Ozaki O, Mimura T, et al. Immediate causes of death in thyroid carcinoma: clinicopathological analysis of 161 fatal cases. J Clin Endocrinol Metab. 1999;84(11):4043-9. 10.1210/jcem.84.11.6115 10566647

[33] 33. Eustatia-Rutten C, Corssmit E, Biermasz N, Pereira A, Romijn J, Smit J. Survival and death causes in differentiated thyroid carcinoma. J Clin Endocrinol Metab. 2006;91(1):313-9. 10.1210/jc.2005-1322 16263822

[34] 34. Lang BH, Wong KP, Cheung CY, Wan KY, Lo C. Evaluating the prognostic factors associated with cancer-specific survival of differentiated thyroid carcinoma presenting with distant metastasis. Ann Surg Oncol. 2013;20:1329-35. 10.1245/s10434-012-2711-x PMC3599207 23104708

[35] 35. Tollefsen HR, Decosse JJ, Hutter RV. Papillary carcinoma of the thyroid. A clinical and pathological study of 70 fatal cases. Cancer. 1964;17:1035-44. 10.1002/1097-0142(196408)17:8<1035::aid-cncr2820170810>3.0.co;2-w 14202591

[36] 36. Smith SA, Hay ID, Goellner JR, Ryan JJ, McConahey WM. Mortality from papillary thyroid carcinoma. A case-control study of 56 lethal cases. Cancer. 1988;62:1381-8. 10.1002/1097-0142(19881001)62:7<1381::aid-cncr2820620724>3.0.co;2-r 3416277

